# Rotational range of motion of elliptical and spherical heads in shoulder arthroplasty: a dynamic biomechanical evaluation

**DOI:** 10.1007/s00402-020-03587-0

**Published:** 2020-08-31

**Authors:** Lukas N. Muench, Alexander Otto, Cameron Kia, Elifho Obopilwe, Mark P. Cote, Andreas B. Imhoff, Knut Beitzel, Augustus D. Mazzocca, Julian Mehl

**Affiliations:** 1 Department of Orthopaedic Surgery, UConn Health, Farmington, CT USA; 2 Department of Orthopaedic Sports Medicine, Technical University of Munich, Munich, Germany; 3 Department of Trauma, Orthopaedic, Plastic and Hand Surgery, University Hospital of Augsburg, Augsburg, Germany; 4 Department of Shoulder Surgery, ATOS Clinic, Cologne, Germany

**Keywords:** Humeral head, Elliptical, Spherical, Prosthesis design, Total shoulder arthroplasty, Hemiarthroplasty, Rotational range of motion

## Abstract

**Introduction:**

Elliptical shape humeral head prostheses have been proposed to reflect a more anatomic shoulder replacement. Its effect on the rotational range of motion (ROM) compared to a standard spherical head is still not understood. The purpose was to investigate if there would be a difference in rotational ROM when comparing elliptical and spherical prosthetic heads in a dynamic shoulder model. The authors hypothesized that the use of elliptical heads would result in significantly more rotational ROM compared to the spherical head design.

**Materials and methods:**

Six fresh-frozen, cadaveric shoulders were evaluated using a dynamic shoulder model. After being tested in the native condition, each specimen underwent 6 conditions in the hemiarthroplasty state: (1) matched-fit spherical head, (2) oversized spherical head, (3) undersized spherical head, (4) matched-fit elliptical head, (5) oversized elliptical head, and (6) undersized elliptical head. Following conversion to total shoulder arthroplasty (TSA), the 6 prior conditions were rerun. Each condition was tested at 0°, 30° and 60° of glenohumeral abduction. Rotational ROM was quantified using 3-dimensional tracking, while dynamically applying alternating forces for internal and external rotation via the rotator cuff tendons.

**Results:**

Elliptical and spherical prosthetic heads showed no significant difference in the degree of the total, internal, and external rotational ROM for both the hemiarthroplasty and TSA state. Conversion from hemiarthroplasty to TSA resulted in less degree of total rotational ROM for both head designs in all abduction positions, without reaching statistical significance. There was a significant decrease in total, internal, and external rotational ROM for both elliptical and spherical heads in every replacement condition, when comparing 0° to 30° and 60° of abduction (*P* < 0.05, respectively).

**Conclusion:**

In a dynamic shoulder model, elliptical and spherical prosthetic head designs showed no significant difference in the degree of the total, internal, and external rotational ROM in both the hemiarthroplasty and TSA state.

**Level of evidence:**

Controlled laboratory study

**Electronic supplementary material:**

The online version of this article (10.1007/s00402-020-03587-0) contains supplementary material, which is available to authorized users.

## Introduction

Shoulder arthroplasty has proven to be a viable treatment option for patients with advanced glenohumeral osteoarthritis (OA), ensuring both pain relief and improved shoulder function [3, 10, 23]. As the native glenohumeral joint has generally been considered to be an articulation of two perfectly spherical components [2, 20, 21, 27], evaluations of shoulder joint kinematics have traditionally been performed assuming the humeral head to be completely spherical in shape [8, 9, 19, 20, 26]. These previous observations are most likely to have inspired current commercially available prosthetic humeral head designs, consequently being completely spherical in shape.

However, several recent anatomic studies have described the humeral head to be more elliptical in shape, rather than a perfect sphere [7, 12, 16]. These studies have reported that the humeral head diverges to elliptical in the anterior–posterior dimension at the periphery of the articular margin, having roughly an 8–12% difference in head radius when comparing frontal and sagittal planes [12, 16]. With the development of glenohumeral OA, initial pathologic deformations are commonly observed at the humeral side [7, 22, 31]. These humeral changes can cause further progression of this elliptical shape, especially in cases of severe disease [7, 22, 31].

As implants resembling the native anatomy may ensure joint kinematics and durability most sufficiently, these findings have questioned if using a spherical prosthesis design is most suitable to replicate the native humeral head [17]. Previous biomechanical studies have shown that a non-spherical prosthetic head more accurately replicated native glenohumeral joint properties in terms of kinematics, translation, and rotational range of motion (ROM) [17, 18]. More importantly, Jun et al. found that the spherical design resulted in less internal rotation at higher abduction angles [17]. This decrease in the high internal rotation may place more stress on the rotator cuff, as the scapulothoracic muscles have to compensate for the lack of glenohumeral rotation. Thus, elliptical heads may allow for a more economical function of the rotator cuff muscles.

However, these previous studies were limited to their static testing design, leaving limited information on how elliptical prosthetic heads would perform in a dynamic shoulder model. Thus, the purpose of this study was to investigate if there would be a difference in the degree of rotational ROM when comparing elliptical and spherical prosthetic heads in a dynamic shoulder model, allowing for evaluation of joint kinematics during external and internal rotation with the forces induced over the rotator cuff muscles. The authors hypothesized that the use of elliptical heads would result in significantly more rotational ROM compared to the spherical head design.

## Materials and methods

Six fresh-frozen, cadaveric shoulders with a mean age of 65 years (range 55–72 years) were used for the study (Science Care Inc., Phoenix, AZ, USA). Prior Institutional Review Board was not required for this study, as de-identified specimens do not constitute human subjects research. All specimens underwent visual and radiographic inspection to exclude those with tears of the rotator cuff tendons and capsule, moderate to severe osteoarthritis, bony defects, or joint contractures.

**Fig. 1:**
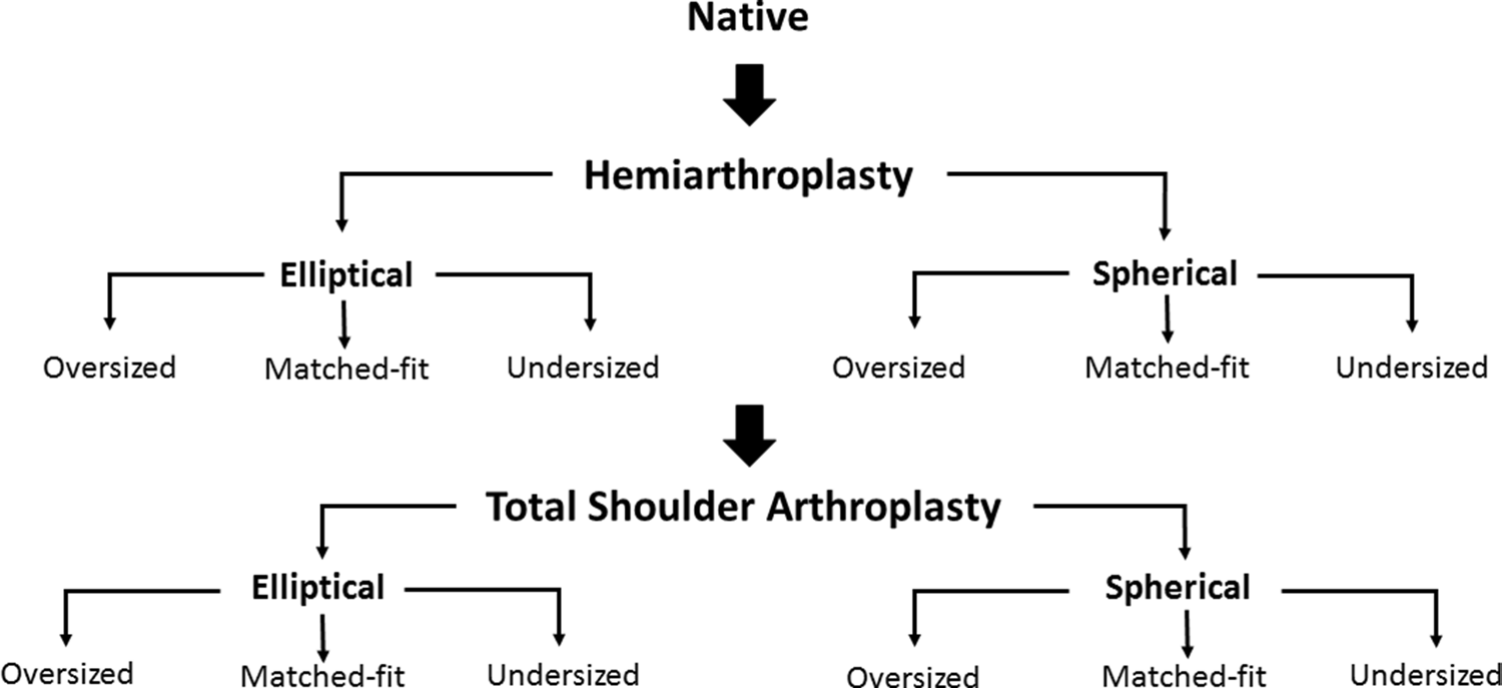
Biomechanical testing flowchart

In total, every specimen underwent 13 different conditions with each specimen being its own control (Fig. 1). First, the specimen was tested in the (1) native state. Following hemiarthroplasty, each shoulder sequentially underwent 6 different conditions: (2) matched-fit spherical head, (3) oversized spherical head, (4) undersized spherical head, (5) matched-fit elliptical head, (6) oversized elliptical head, and (7) undersized elliptical head. The hemiarthroplasty was then converted to a total shoulder arthroplasty (TSA) and specimens underwent the 6 above-mentioned conditions again (8 to 13).

To avoid selection bias, the order of tested prosthetic head shapes, sizes, as well as glenohumeral abduction positions (0°, 30°, 60°) were randomly assigned using computer software within the states of hemiarthroplasty and TSA. The specimens remained in the shoulder simulator throughout all testing and surgical repairs.

### Specimen preparation

Specimen preparation was performed according to a previously described method [5]. After having been thawed overnight at room temperature, specimens were dissected free of skin, subcutaneous tissue and muscles. Rotator cuff muscles and the coracoacromial ligament were carefully preserved. At the deltoid tuberosity, the anterior, middle, and posterior portions of the deltoid tendon were detached from the muscle belly and preserved. Anchor loops were sutured to the tendinous insertions using a locking running stitch (No. 2 FiberWire, Arthrex Inc., Naples, FL, USA), allowing for attachment of each of the three deltoid heads to an individual shoulder simulator actuator. At the scapular site, the rotator cuff muscles (supraspinatus (SSP), subscapularis (SSC), infraspinatus (ISP), and teres minor (TM)) were released off and separated from the underlying capsule, while meticulously preventing disruption of the tissue. ISP and TM were simulated as one unit [15].

Subsequently, the individual rotator cuff tendons were sutured to pulley-straps using No. 2 FiberWire to avoid pull-through during load application. After the scapular body was placed in a custom rectangular box with the medial border aligned perpendicular to the ground and the glenoid tilted 10° superiorly, bone cement was poured in to ensure proper fixation [11, 30]. A steel rod was cemented into the distal humerus and loaded with 1.7 kg, 30 cm distal from the center of the humeral head. This represented a constant moment arm for each tested shoulder [15, 30]. The glenohumeral joint capsule was vented by opening the rotator interval, to prevent changes during testing.

### Testing setup

**Fig. 2:**
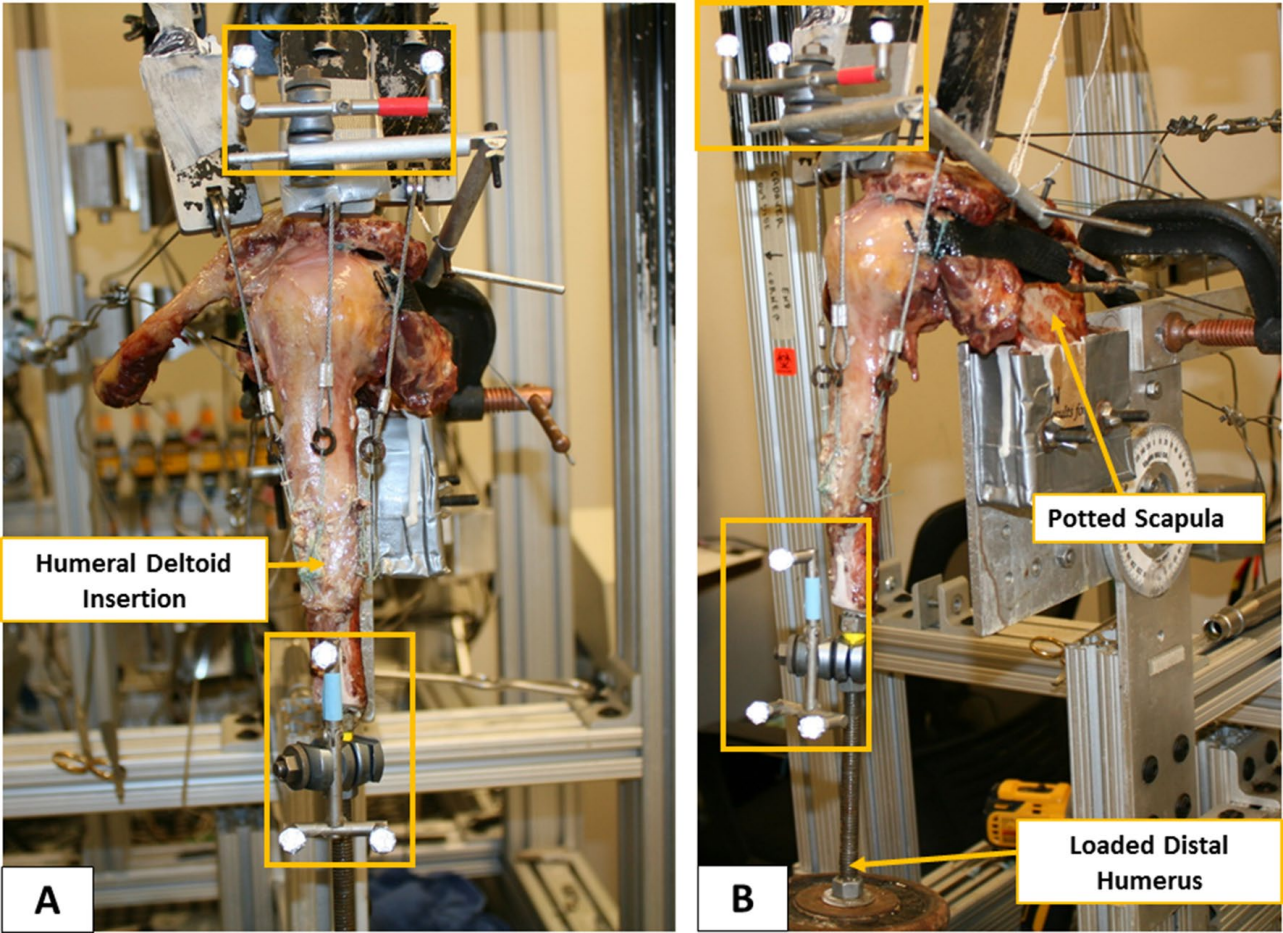
Biomechanical testing setup in the anterior (**a**) and lateral (**b**) view, including the triads (orange boxes) for optical motion tracking

A standard dynamic shoulder model was utilized, adapted from previously validated cadaveric shoulder studies (Fig. 2) [4, 5, 11, 15, 24, 25, 30]. The shoulder simulator consisted of up to six linear screw-driven actuators (Bimba, Monee, IL, USA) connected to 444 N load cells (Futek, Irvine, CA, USA). A universal strain gauge signal conditioner (Futek Model CSG110) was linked to a panel mount display (Futek Model IMP 650), and a test and measurement software (Sensit V2.5.1.0, Futek, Irvine, CA, USA) was used for load cells data acquisition in real time [5].

To most accurately represent the resting position of the scapula, the specimen was mounted to the simulator on a 6 degrees-of-freedom jig with the scapula in 10° of anteflexion [30]. This, in combination with the 10° superior tilt of the glenoid, resulted in a 110° angle between the scapular spine and vertical axis [30]. The anatomic lines of action of the three portions of the deltoid, SSC and ISP/TM unit were routed using custom 7 mm-diameter frictionless pulleys. The cable attached to the SSP tendon was aligned with a tilt of 10° to the horizontal [30]. Mimicking the native force vectors, the pulley for the anterior deltoid was placed over the tip of the coracoid process, approximately 5 mm anteriorly to the anterolateral corner of the acromion. The middle deltoid pulley was routed over a point 5 mm posteriorly to the anterolateral corner of the acromion, whereas the posterior deltoid pulley was placed at the posterolateral edge of the acromion in line with the scapular spine [30].

Prior to testing, four infrared cameras (Vero v1.3, Vicon Motion Capture Systems, Centennial, CO, USA) were mounted around the shoulder simulator to cover a 180° field of view. A stationary triad, consisting of 3 optical markers, was placed on the acromion with its center being in line with the pulley of the middle deltoid. A second moving triad was mounted to the humeral shaft with its longitudinal axis being in line with the center of the stationary triad placed on the acromion.

In a displacement-controlled setting, computer software (SiNet Hub Programmer V1.29; Applied Motion Products, Inc., CA, USA) was utilized to generate custom motion profiles for the individual actuators of the SSP as well as the anterior, middle, and posterior deltoid separately for each specimen. 3-dimensional (3D) motion tracking (Vicon Nexus 2.8, Vicon Motion Capture Systems, Oxford, UK) and four infrared cameras (Vicon Vero v1.3) with a frame rate of 250 Hz and a position accuracy of 0.01 mm and 0.1 degrees, recorded each motion profile with the arm being abducted in neutral rotation from 0° to 60° in the scapular plane with the scapula fixed, corresponding to approximately 90° of total shoulder abduction. For calculation of these custom motion profiles, the SSC and ISP/TM unit were loaded statically with a 1.36 kg hanging weight, allowing for a balanced abduction motion [25]. To generate reliable data of applied forces, each motion cycle was repeated three times [4]. To maintain joint centering at the resting position, 10 N were applied to the SSP as well as the anterior, middle, and posterior deltoid, respectively [29].

Once an anatomic abduction motion profile was created, the specimen was stopped at 30° and 60° of abduction, respectively. Forces of the SSP as well as the anterior, middle, and posterior deltoid were obtained from the load cells in the 30° and 60° abduction position. Subsequently, the setting was switched from displacement-controlled to force-controlled (LabVIEW 2018 (32-bit), National Instruments, Austin, TX, USA). The SSC and ISP/TM unit were also attached to corresponding actuators, each with 10 N being applied at the resting position [29].

### Dynamic biomechanical testing

Each condition (1 to 13) was tested in 3 different degrees of glenohumeral abduction (0, 30, 60) [17]. Every testing cycle started with the specimen in its resting position (0° of abduction, neutral rotation). Forces of the SSP, anterior, middle, and posterior deltoid collected from the created motion profile were then implemented into the software, as seeking force values to obtain glenohumeral abduction angles of 30° and 60°, respectively. Subsequently, alternating forces were applied to the SSC tendon (60 N) for dynamic internal rotation and the ISP/TM tendon unit (40 N) for dynamic external rotation in a 3:2 ratio [32]. Each cycle was repeated three times. The optical markers were tracked and recorded by infrared cameras, allowing for the accurate evaluation of rotational ROM using computer software (Vicon Nexus 2.8 and Vicon proCalc 1.2.1, Vicon Motion Capture Systems). Additional live-video fluoroscopy was performed using a mobile mini C-arm (MiniView 6800 Mobile Imaging System, GE OEC Medical Systems Inc., UT, USA), to radiographically confirm accurate implant placement and centered rotational motion.

### Surgical technique

The hemiarthroplasty and TSA were performed based on a previously described technique, using an anatomic stemless implant (Eclipse system, Arthrex Inc., Naples, FL, USA) [6, 10, 28]. First, the rotator interval was extended and the coracohumeral ligament was cut at its insertion at the coracoid process. Next, a juxta-glenoidal capsulotomy was made in direction to the 6 o’clock position, while preserving the anterior portion of the inferior glenohumeral ligament (aIGHL). The subscapularis tendon was preserved to ensure a standard approach and preclude implementation of another variable, potentially causing alterations during dynamic testing.

**Fig. 3:**
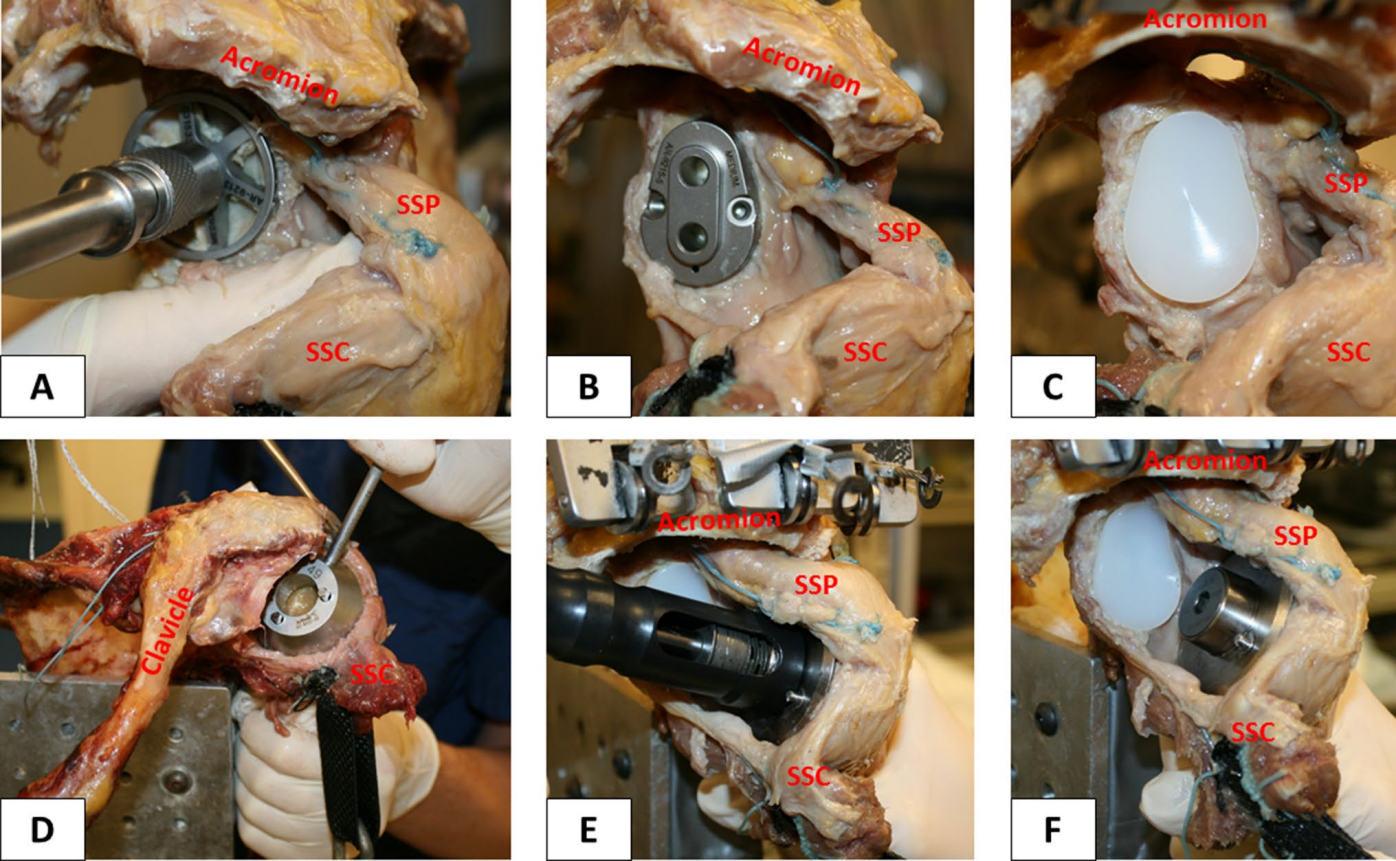
Demonstrating the surgical technique. Reaming the articular surface of the glenoid (**a**). Glenoid guide placed on the central axis of the exposed glenoid surface (**b**). Inserted keeled glenoid implant (**c**). Determination of trunnion size on the resected humeral head (**d**). Insertion of the hollow screw to secure trunnion (**e**). Trunnion is additionally secured with a small, protruding spike to allow for easy switching of the prosthetic heads (**f**). SSP = supraspinatus; SSC = subscapularis

Oriented along the specimen’s anatomic retrotorsion, two 1.6 mm K-wires were pre-drilled in line with the desired resection plane, exiting the opposite cortex at the boundary of the articular cartilage. Guided by the two K-wires, an osteotomy was performed using an oscillating saw. After measuring the anterior–posterior dimension of the resected humeral head, the size of the baseplate (trunnion) was determined (Fig. 3d). The trunnion was then fixed to the resected humeral neck and a hollow screw was inserted (Fig. 3e). The custom made trunnion used for this study was additionally secured with a small, protruding spike, to allow for easy switching of prosthetic head shapes and sizes (Fig. 3f).

**Fig. 4:**
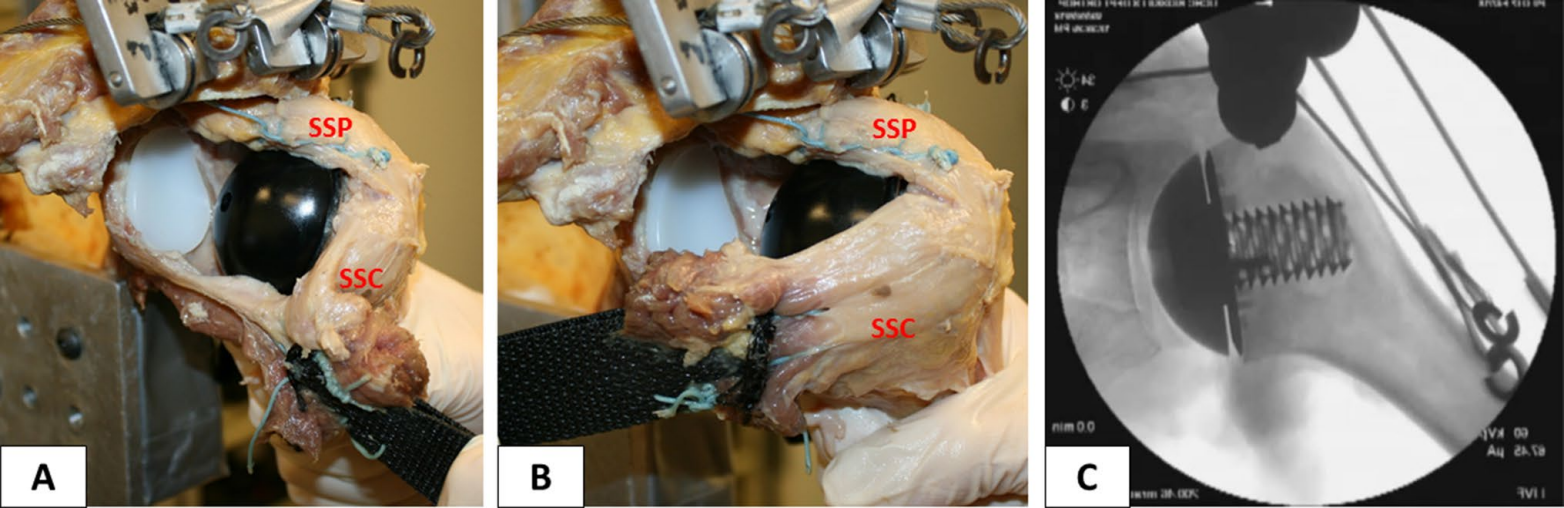
Completed stemless total shoulder arthroplasty in the lateral view with (**a**) and without (**b**) retracted subscapularis muscle for better visualization. Fluoroscopy image of implanted prosthesis (**c**)

Conversion to TSA was performed using a keeled glenoid system (Univers II, Arthrex Inc., Naples, FL, USA) (Fig. 3a–c). A glenoid guide was placed on the central axis of the exposed articular surface of the glenoid, with the guide handle being oriented in line with the anatomic slope of the anterior neck. Following preparation, a keeled glenoid implant was inserted in the created slot and impacted (Fig. 4).

### Humeral head prosthetic design

Both elliptical and spherical humeral head prostheses used in this study were custom made (Arthrex Inc., Naples, FL, USA). The designs, including equations for dimension width, the radius of curvature, and height, were based on previously published studies [13, 14]. A small hole in the undersurface allowed for securely placing the humeral head prosthesis on the protruding spike of the Eclipse trunnion, avoiding rotation of the head prosthesis during testing and allowing for easily switching heads. Oversized and undersized was defined as one size larger or smaller than the matched-fit head size.

### Statistical analysis

A power analysis was carried out to determine detectable differences in rotational ROM, using standard deviations estimated from the literature as well as pilot data from our laboratory [17]. Assuming a common standard deviation of 5.5 degrees, the sample size of 6 specimens would provide 80% power to detect a 10-degree difference in rotational ROM at an α level of 0.05. Descriptive statistics, including mean and standard deviations, were calculated to characterize the study groups. A mixed-effects linear regression model was used to examine differences in ROM between the elliptical and spherical prosthetic head designs at varying degrees of abduction and implant fits. The distributions of the model residuals were examined to ensure large deviations from normality were not present. A random intercept was used to account for the testing of specimens in varying conditions. Mean differences were reported along with corresponding 95% confidence intervals (CI). A *P* value < 0.05 was set to be statistically significant. All statistical analyses were conducted with Stata 15 software (StataCorp. 2017. Stata Statistical Software: Release 15. College Station, TX: StataCorp LLC).

## Results

**Fig. 5:**
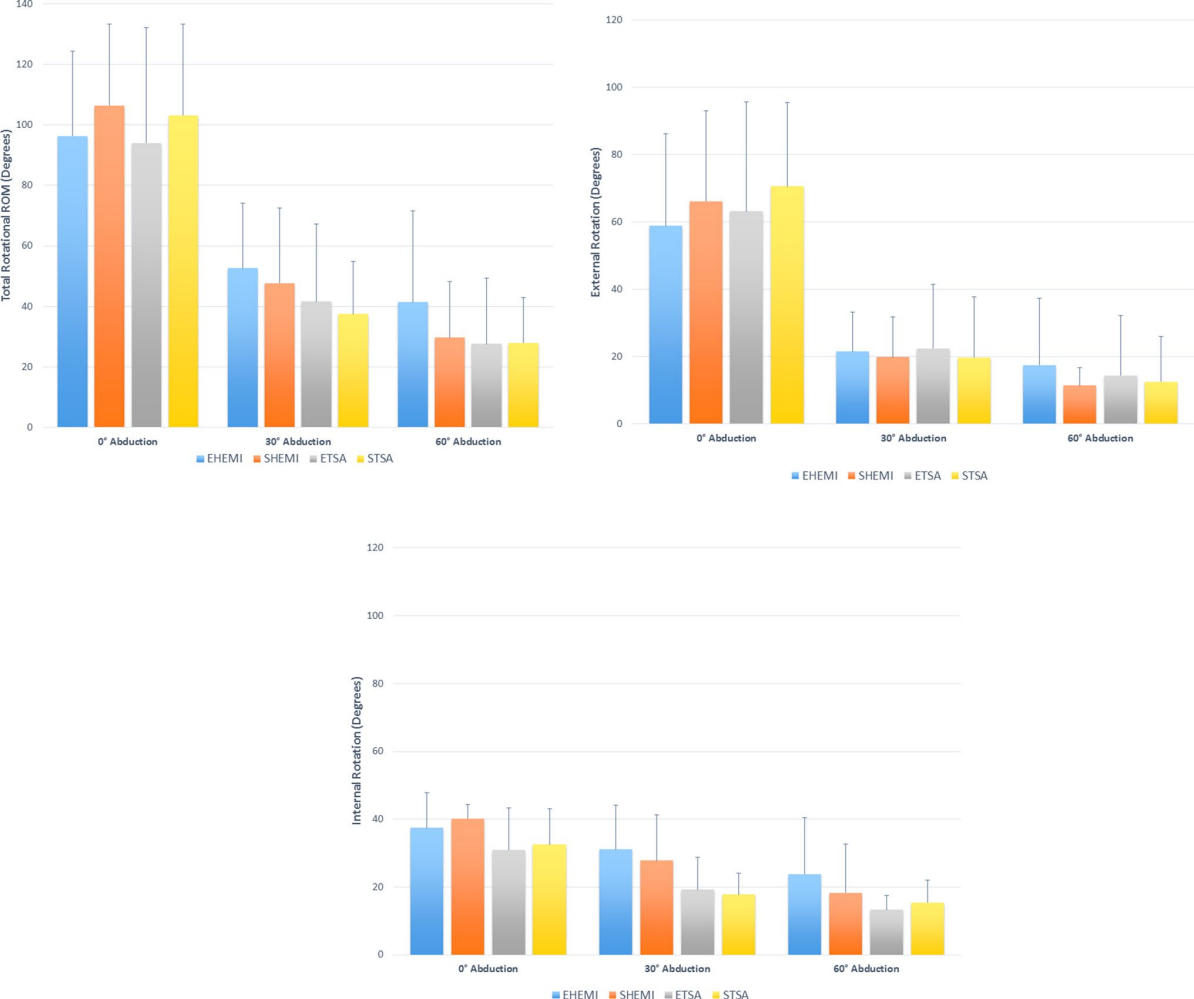
Demonstrating a degree of total (**a**), external (**b**), and internal (**c**) rotational range of motion (ROM) in 0°, 30°, and 60° of glenohumeral abduction. EHEMI = elliptical hemiarthroplasty; SHEMI = spherical hemiarthroplasty; ETSA = elliptical total shoulder arthroplasty; STSA = spherical total shoulder arthroplasty

Overall, elliptical and spherical prosthetic heads showed no significant difference in the degree of total, internal, and external rotational ROM in both the hemiarthroplasty and TSA state (*P* > 0.05, respectively) (Fig. 5a–c). Absolute mean values of rotational ROM are demonstrated in Table 1. With regard to total rotational ROM using matched-fit head sizes in the hemiarthroplasty state, the difference between the elliptical and spherical head averaged 10.1° (*P* = 0.431; CI − 15.0 to 35.2) at 0° of abduction in favour of the spherical design, whereas in 30° and 60° of abduction the mean difference was − 4.9° (*P* = 0.701; CI − 30.0 to 20.2) and − 11.7° (*P* = 0.36; CI − 36.8 to 13.4), respectively, favouring the elliptical design. On average in the TSA state, 0° of abduction showed a greater ROM for the spherical head (mean difference: 9.1°; *P* = 0.476; CI − 16.0 to 34.2), while the 30° position favoured the elliptical head (mean difference: − 4.1°; *P* = 0.749; CI − 29.2 to 21.0). In 60° of abduction, both designs showed similar values (mean difference: 0.4°; *P* = 0.978; CI − 24.8 to 25.5).

**Table 1:** Demonstrating the degree of axial rotation at various abduction angles

		0° Abduction			30° Abduction			60° Abduction		
		Total ROM	ER	IR	Total ROM	ER	IR	Total ROM	ER	IR
Matched-fit										
EHEMI	Mean	96.3	58.9	37.4	52.7	21.6	31.1	41.4	17.5	23.8
	sd	28.1	27.4	10.4	21.6	11.6	13.1	30.3	19.9	16.6
SHEMI	Mean	106.4	66.2	40.2	47.7	19.9	27.8	29.6	11.4	18.3
	sd	26.9	26.8	4.1	24.9	11.9	13.5	18.6	5.4	14.5
ETSA	Mean	94.0	63.2	30.9	41.6	22.4	19.2	27.6	14.4	13.2
	sd	38.0	32.6	12.5	25.6	19.0	9.7	21.8	17.8	4.4
STSA	Mean	103.2	70.7	32.5	37.5	19.7	17.8	28.0	12.5	15.5
	sd	30.2	24.8	10.6	17.4	18.1	6.3	15.0	13.4	6.6
Oversized										
EHEMI	Mean	106.5	66.4	40.1	50.6	22.7	27.9	30.5	9.9	20.5
	sd	23.6	25.0	8.8	31.0	18.4	14.5	24.4	8.0	16.9
SHEMI	Mean	109.0	70.0	39.0	45.6	17.4	28.2	28.3	11.1	17.2
	sd	34.7	32.5	12.0	26.6	13.2	16.4	23.4	7.5	17.1
ETSA	Mean	102.8	70.2	32.6	38.2	17.6	20.6	27.4	15.3	12.1
	sd	23.3	26.6	7.4	26.7	20.1	11.3	23.1	17.6	7.9
STSA	Mean	102.3	68.8	33.5	32.2	14.6	17.6	26.8	15.8	11.1
	sd	30.9	27.7	13.8	16.7	12.1	9.1	27.3	16.2	11.5
Undersized										
EHEMI	Mean	93.2	57.3	35.9	46.3	21.5	24.9	33.2	13.0	20.2
	sd	29.8	26.9	14.4	29.7	17.9	13.2	22.5	9.0	15.2
SHEMI	Mean	89.6	57.0	32.6	47.0	17.5	29.5	36.9	13.0	23.9
	sd	21.3	22.4	8.2	26.0	12.0	15.1	24.1	11.6	15.7
ETSA	Mean	98.7	70.5	28.2	41.1	24.6	16.5	27.2	16.4	10.7
	sd	26.4	26.5	10.6	27.9	21.8	8.4	24.2	19.1	6.6
STSA	Mean	100.2	66.6	33.6	39.0	18.4	20.6	28.5	13.3	15.2
	sd	26.5	23.9	9.9	20.6	16.9	8.4	20.9	16.5	6.3

*SD* standard deviation, *ROM* range of motion, *ER* external rotation, *IR* internal rotation, *EHEMI* elliptical hemiarthroplasty, *ETSA* elliptical total shoulder arthroplasty, *SHEMI* spherical hemiarthroplasty, *STSA* spherical total shoulder arthroplasty

Conversion from hemiarthroplasty to TSA consistently resulted in less degree of total rotational ROM for both head designs in all abduction angles, without reaching statistical significance (*P* > 0.05, respectively) (Fig. 5a–c). Regarding elliptical heads, the hemiarthroplasty state showed significantly more internal rotation in 30° (mean difference: − 11.9°; *P* = 0.019; CI − 21.9 to − 1.9) and 60° (mean difference: − 10.6°; *P* = 0.038; CI − 20.6 to − 0.6) of abduction, when compared to TSA. An overview of comparisons between replacement conditions is presented in supplemental file 1.

A significant decrease in degree of total, internal, and external rotational ROM was found for both matched-fit elliptical and spherical heads in the hemiarthroplasty and TSA state, when comparing the resting position to 30° and 60° of abduction (*P* < 0.05, respectively). We found no significant difference in rotational ROM between abduction angles of 30° and 60° in all replacement conditions (*P* > 0.05, respectively). When comparing matched-fit, oversized, and undersized heads within each replacement condition, we generally found that under-sizing the prosthetic head resulted in less degree of rotational ROM. However, these findings were only statistically significant in 0° of abduction in the hemiarthroplasty state when comparing the undersized spherical head with the matched-fit (mean difference: − 16.9°; *P* = 0.022; CI − 31.2 to − 2.5) or oversized (mean difference: − 19.5°; *P* = 0.008; CI − 33.9 to − 5.1) head. All other comparisons were not significant and are presented in supplemental file 2.

## Discussion

The most important finding of this study was that elliptical and spherical prosthetic heads showed no significant difference in degree of total, internal, and external rotational ROM in both the hemiarthroplasty and TSA state when using a dynamic shoulder model. In addition, this study found a general decrease in the degree of rotational ROM with higher glenohumeral abduction angles for both elliptical and spherical heads in hemi- and total shoulder arthroplasty.

As several anatomic studies have described the humeral head to be more elliptical in shape rather than a perfect sphere [7, 12, 16], prosthetic designs resembling the native anatomy have recently garnered more interest. A biomechanical study by Jun et al. compared non-spherical and spherical heads prosthesis to the native humeral head in shoulders that underwent hemiarthroplasty [17]. The authors found no significant difference in rotational ROM between the non-spherical prosthetic head and native head, whereas the spherical head design led to significant decreases, especially in internal rotation in the scapular plane [17]. A difference between the two prosthetic head designs was only observed in 30° and 60° of abduction in the scapular plane, with the non-spherical design achieving significantly more internal rotation [17]. However, this study was limited to its static testing design and kinematics were not evaluated following conversion to TSA [17]. In their static model, Jun et al. applied the axial rotational forces over the humerus using a torque wrench [17]. In contrast, our testing setup allowed for dynamically inducing the required rotational forces over the rotator cuff muscles (subscapularis and infraspinatus/teres minor), which simulates an in vivo shoulder setting more accurately.

Despite our findings not being statistically significant, a trend was observed with spherical heads reaching more rotational ROM in the resting position, whereas elliptical heads had greater motion at higher abduction angles. The elliptical head design is based on comprising less material at the anterior and posterior side, which may result in less translation on the glenoid caused by the anterior and posterior rotator cuff muscles pushing the implant away during rotation. This may be especially of importance at higher abduction angles with the glenohumeral joint being more constraint. However, the maximum averaged difference in total rotational ROM was only 11.7°, favouring the elliptical design at 60° of abduction in the hemiarthroplasty state. This was similar to the findings by Jun et al. [17] who reported a mean difference of 7.1° when comparing non-spherical and spherical heads. While the previous authors found statistical significance, the clinical relevance of this change is unlikely to impair function [17]. However, the elliptical design may play a greater role in the longevity of the implant, rather than the immediate postoperative gains in motion.

Previous cadaveric studies have suggested that glenohumeral mechanics can be significantly altered with a change of 4–5 mm in the articulating surface during shoulder arthroplasty [9, 18]. This has raised concerns for the spherical design, as this magnitude of mismatch has been shown with this type of prosthesis [13, 14]. Humphrey et al. recently compared spherical to elliptical prosthetic heads in a 3-dimensional model [13]. The authors found regardless of head size, a spherical prosthesis was only capable of matching the native head (within 3 mm) in 41–78% of cases, compared 96–100% for elliptical designs [13]. Theoretically, this suggested that the elliptical heads could decrease costs by requiring fewer sizes in sets, and improve clinical results in up to 60% of more patients [13].

Clinical results of the elliptical prosthetic design have yet to be reported. Previously published clinical studies reporting on patients who underwent hemiarthroplasty or TSA using the same spherical stemless prosthesis design showed sufficient functional and radiographic results after a mean follow-up of up to 9 years [6, 10, 28]. One recent study found implant survival to be 93.5% at 8 years using a spherical design, with continued significant improvement in VAS and Constant scores [1]. The various morphologies of the humeral head, especially in the setting of osteoarthritis, may make availability of both prostheses important for clinical outcomes.

The magnitude of this described elliptical shape of the native humeral head may be increased in further progression of high-grade OA. This was confirmed clinically by Habermeyer et al. [7], who found that 82.2% of patients who underwent anatomic shoulder arthroplasty for treatment of primary OA had an aspherical humeral head shape. These observations also implied that 17.8% of the evaluated humeral heads were still spherical in shape, bringing greater confusion as to which patient would most benefit more from a spherical head design rather than from an elliptical one. Thus, these inconsistencies in the anatomy of each individual specimen, with the humeral head being either more spherical or elliptical in shape and differing tightness of the glenohumeral joint capsule, may explain the relatively large variations of differences between spherical and elliptical heads, without reaching significance in favour of one of the two head designs.

There were several limitations to this study. Humeral head prosthetic design may show a different effect in vivo when compared to observations during laboratory cadaveric testing. Even though a dynamic shoulder model was used, this testing design focused on dynamically evaluating rotational ROM only at various positions of glenohumeral abduction in the scapular plane, with measurements in other motion planes, such as flexion, not being investigated. In addition, substantial muscles of the shoulder girdle known to influence shoulder kinematics, such as the latissimus dorsi or pectoralis major, were not taken into account. Another limitation of the study’s testing design is the elimination of scapulothoracic motion by fixing the scapula. However, this was necessary to securely mount the specimen to the simulator. Besides, this allowed for ensuring a standardized orientation of the glenoid and resting position of the tested specimen. Moreover, our shoulder model was not able to account for differing tightness of the glenohumeral joint capsule, which may have caused alterations in rotational kinematics. Lastly, the native rotational ROM results were excluded from analysis, given the fundamental differences in testing setup when analysing an intact shoulder. During surgical replacement, the anteroinferior capsule was opened while preserving the aIGHL, to accurately visualize the resection plane of the humeral head. As switching of prosthetic head shapes and sizes had to be frequently performed during testing, a capsular repair was infeasible, potentially increasing external rotation.

## Conclusion

In a dynamic shoulder model, elliptical and spherical prosthetic head designs showed no significant difference in the degree of the total, internal, and external rotational range of motion in both the hemiarthroplasty and total shoulder arthroplasty state.

## Electronic supplementary material

Below is the link to the electronic supplementary material.

Supplementary file1 (DOCX 20 kb)

Supplementary file2 (DOCX 20 kb)
