# Supplementary material for: Rotational range of motion of elliptical and spherical heads in shoulder arthroplasty: a dynamic biomechanical evaluation
Source: Arch Orthop Trauma Surg. 2020 Aug 31;142(1):67–76. doi: 10.1007/s00402-020-03587-0 (PMC8732933; doi:10.1007/s00402-020-03587-0)
Supplement: Supplementary file 1 — Supplementary file1 (DOCX 20 kb) [file 402_2020_3587_MOESM1_ESM.docx]

|  |  | **Matched-fit** | | | | **Oversized** | | | | **Undersized** | | | | |
| --- | --- | --- | --- | --- | --- | --- | --- | --- | --- | --- | --- | --- | --- | --- |
|  | **Comparison** | **Difference** | **P value** | **95% CI** | | **Difference** | **P value** | **95% CI** | | **Difference** | **P value** | **95% CI** | |  |
| **Total ROM** | ETSA@0 vs EHEMI@0 | -2.3 | 0.860 | -27.4 | 22.8 | -3.7 | 0.779 | -29.4 | 22.1 | 5.5 | 0.647 | -18.0 | 28.9 |  |
|  | SHEMI@0 vs EHEMI@0 | 10.1 | 0.431 | -15.0 | 35.2 | 2.6 | 0.845 | -23.2 | 28.3 | -3.6 | 0.762 | -27.1 | 19.8 |  |
|  | STSA@0 vs ETSA@0 | 9.1 | 0.476 | -16.0 | 34.2 | -0.5 | 0.973 | -26.2 | 25.3 | 1.6 | 0.895 | -21.9 | 25.0 |  |
|  | STSA@0 vs SHEMI@0 | -3.2 | 0.801 | -28.3 | 21.9 | -6.7 | 0.610 | -32.4 | 19.0 | 10.7 | 0.372 | -12.8 | 34.1 |  |
|  | ETSA@30 vs EHEMI@30 | -11.1 | 0.388 | -36.2 | 14.0 | -12.4 | 0.345 | -38.1 | 13.3 | -5.2 | 0.663 | -28.7 | 18.2 |  |
|  | SHEMI@30 vs EHEMI@30 | -4.9 | 0.701 | -30.0 | 20.2 | -5.0 | 0.705 | -30.7 | 20.8 | 0.7 | 0.957 | -22.8 | 24.1 |  |
|  | STSA@30 vs ETSA@30 | -4.1 | 0.749 | -29.2 | 21.0 | -6.0 | 0.648 | -31.7 | 19.7 | -2.2 | 0.856 | -25.6 | 21.3 |  |
|  | STSA@30 vs SHEMI@30 | -10.3 | 0.424 | -35.4 | 14.9 | -13.4 | 0.306 | -39.2 | 12.3 | -8.0 | 0.502 | -31.5 | 15.4 |  |
|  | ETSA@60 vs EHEMI@60 | -13.7 | 0.284 | -38.8 | 11.4 | -3.0 | 0.816 | -28.8 | 22.7 | -6.0 | 0.616 | -29.4 | 17.4 |  |
|  | SHEMI@60 vs EHEMI@60 | -11.7 | 0.360 | -36.8 | 13.4 | -2.2 | 0.868 | -27.9 | 23.6 | 3.8 | 0.753 | -19.7 | 27.2 |  |
|  | STSA@60 vs ETSA@60 | 0.4 | 0.978 | -24.8 | 25.5 | -0.6 | 0.966 | -26.3 | 25.2 | 1.3 | 0.911 | -22.1 | 24.8 |  |
|  | STSA@60 vs SHEMI@60 | -1.7 | 0.897 | -26.8 | 23.4 | -1.4 | 0.913 | -27.2 | 24.3 | -8.4 | 0.481 | -31.9 | 15.0 |  |
|  |  |  |  |  |  |  |  |  |  |  |  |  |  |  |
| **ER** | ETSA@0 vs EHEMI@0 | 4.3 | 0.677 | -16.0 | 24.6 | 3.8 | 0.716 | -16.6 | 24.2 | 13.2 | 0.183 | -6.2 | 32.5 |  |
|  | SHEMI@0 vs EHEMI@0 | 7.4 | 0.478 | -13.0 | 27.7 | 3.6 | 0.727 | -16.8 | 24.0 | -0.4 | 0.972 | -19.7 | 19.0 |  |
|  | STSA@0 vs ETSA@0 | 7.5 | 0.471 | -12.8 | 27.8 | -1.4 | 0.896 | -21.8 | 19.0 | -3.9 | 0.696 | -23.2 | 15.5 |  |
|  | STSA@0 vs SHEMI@0 | 4.4 | 0.669 | -15.9 | 24.7 | -1.2 | 0.907 | -21.6 | 19.2 | 9.7 | 0.329 | -9.7 | 29.0 |  |
|  | ETSA@30 vs EHEMI@30 | 0.9 | 0.933 | -19.4 | 21.2 | -5.1 | 0.624 | -25.5 | 15.3 | 3.2 | 0.750 | -16.2 | 22.5 |  |
|  | SHEMI@30 vs EHEMI@30 | -1.7 | 0.873 | -22.0 | 18.7 | -5.3 | 0.614 | -25.7 | 15.2 | -4.0 | 0.687 | -23.4 | 15.4 |  |
|  | STSA@30 vs ETSA@30 | -2.7 | 0.794 | -23.0 | 17.6 | -3.0 | 0.774 | -23.4 | 17.4 | -6.3 | 0.526 | -25.6 | 13.1 |  |
|  | STSA@30 vs SHEMI@30 | -0.2 | 0.986 | -20.5 | 20.1 | -2.8 | 0.785 | -23.2 | 17.6 | 0.9 | 0.930 | -18.5 | 20.2 |  |
|  | ETSA@60 vs EHEMI@60 | -3.1 | 0.762 | -23.4 | 17.2 | 5.4 | 0.607 | -15.1 | 25.8 | 3.4 | 0.728 | -15.9 | 22.8 |  |
|  | SHEMI@60 vs EHEMI@60 | -6.1 | 0.554 | -26.4 | 14.2 | 1.2 | 0.911 | -19.2 | 21.6 | 0.0 | 0.997 | -19.3 | 19.4 |  |
|  | STSA@60 vs ETSA@60 | -1.9 | 0.856 | -22.2 | 18.4 | 0.5 | 0.963 | -19.9 | 20.9 | -3.1 | 0.753 | -22.5 | 16.3 |  |
|  | STSA@60 vs SHEMI@60 | 1.1 | 0.914 | -19.2 | 21.4 | 4.7 | 0.654 | -15.7 | 25.1 | 0.3 | 0.977 | -19.1 | 19.7 |  |
|  |  |  |  |  |  |  |  |  |  |  |  |  |  |  |
| **IR** | ETSA@0 vs EHEMI@0 | -6.6 | 0.197 | -16.6 | 3.4 | -7.5 | 0.177 | -18.3 | 3.4 | -7.7 | 0.123 | -17.4 | 2.1 |  |
|  | SHEMI@0 vs EHEMI@0 | 2.7 | 0.590 | -7.3 | 12.8 | -1.1 | 0.847 | -11.9 | 9.8 | -3.3 | 0.511 | -13.0 | 6.5 |  |
|  | STSA@0 vs ETSA@0 | 1.7 | 0.744 | -8.3 | 11.7 | 0.9 | 0.868 | -9.9 | 11.7 | 5.5 | 0.273 | -4.3 | 15.2 |  |
|  | STSA@0 vs SHEMI@0 | -7.7 | 0.133 | -17.7 | 2.3 | -5.5 | 0.321 | -16.3 | 5.3 | 1.0 | 0.835 | -8.7 | 10.8 |  |
|  | ETSA@30 vs EHEMI@30 | -11.9 | 0.019* | -21.9 | -1.9 | -7.3 | 0.186 | -18.1 | 3.5 | -8.4 | 0.093 | -18.1 | 1.4 |  |
|  | SHEMI@30 vs EHEMI@30 | -3.3 | 0.522 | -13.3 | 6.7 | 0.3 | 0.959 | -10.5 | 11.1 | 4.6 | 0.352 | -5.1 | 14.4 |  |
|  | STSA@30 vs ETSA@30 | -1.4 | 0.784 | -11.4 | 8.6 | -3.0 | 0.585 | -13.8 | 7.8 | 4.1 | 0.410 | -5.7 | 13.9 |  |
|  | STSA@30 vs SHEMI@30 | -10.1 | 0.049 | -20.1 | -0.1 | -10.6 | 0.055 | -21.4 | 0.2 | -8.9 | 0.074 | -18.7 | 0.9 |  |
|  | ETSA@60 vs EHEMI@60 | -10.6 | 0.038* | -20.6 | -0.6 | -8.4 | 0.128 | -19.2 | 2.4 | -9.4 | 0.058 | -19.2 | 0.3 |  |
|  | SHEMI@60 vs EHEMI@60 | -5.6 | 0.274 | -15.6 | 4.4 | -3.4 | 0.544 | -14.2 | 7.5 | 3.7 | 0.453 | -6.0 | 13.5 |  |
|  | STSA@60 vs ETSA@60 | 2.2 | 0.662 | -7.8 | 12.2 | -1.1 | 0.849 | -11.9 | 9.8 | 4.5 | 0.371 | -5.3 | 14.2 |  |
|  | STSA@60 vs SHEMI@60 | -2.8 | 0.586 | -12.8 | 7.2 | -6.1 | 0.270 | -16.9 | 4.7 | -8.7 | 0.080 | -18.5 | 1.0 |  |
