# Supplementary material for: Rotational range of motion of elliptical and spherical heads in shoulder arthroplasty: a dynamic biomechanical evaluation
Source: Arch Orthop Trauma Surg. 2020 Aug 31;142(1):67–76. doi: 10.1007/s00402-020-03587-0 (PMC8732933; doi:10.1007/s00402-020-03587-0)
Supplement: Supplementary file 2 — Supplementary file2 (DOCX 20 kb) [file 402_2020_3587_MOESM2_ESM.docx]

|  |  | **0° Abduction** | | | | **30° Abduction** | | | | **60° Abduction** | | | | |
| --- | --- | --- | --- | --- | --- | --- | --- | --- | --- | --- | --- | --- | --- | --- |
|  | **Comparison** | **Difference** | **P value** | **95% CI** | | **Difference** | **P value** | **95% CI** | | **Difference** | **P value** | **95% CI** | |  |
| **Total ROM** | EHEMI_over vs EHEMI_matched | 10.2 | 0.166 | -4.2 | 24.5 | -2.1 | 0.646 | -11.1 | 6.9 | -10.9 | 0.032* | -20.8 | -1.0 |  |
|  | EHEMI_under vs EHEMI_matched | -3.1 | 0.669 | -17.5 | 11.2 | -6.3 | 0.168 | -15.3 | 2.7 | -8.2 | 0.106 | -18.1 | 1.8 |  |
|  | EHEMI_under vs EHEMI_over | -13.3 | 0.07 | -27.7 | 1.1 | -4.2 | 0.357 | -13.2 | 4.8 | 2.7 | 0.592 | -7.2 | 12.7 |  |
|  | ETSA_over vs ETSA_matched | 8.8 | 0.233 | -5.6 | 23.1 | -3.4 | 0.453 | -12.4 | 5.5 | -0.2 | 0.966 | -10.2 | 9.7 |  |
|  | ETSA_under vs ETSA_matched | 4.6 | 0.529 | -9.8 | 19.0 | -0.5 | 0.919 | -9.4 | 8.5 | -0.4 | 0.929 | -10.4 | 9.5 |  |
|  | ETSA_under vs ETSA_over | -4.1 | 0.573 | -18.5 | 10.2 | 3.0 | 0.517 | -6.0 | 11.9 | -0.2 | 0.963 | -10.2 | 9.7 |  |
|  | SHEMI_over vs SHEMI_matched | 2.6 | 0.72 | -11.7 | 17.0 | -2.2 | 0.639 | -11.1 | 6.8 | -1.4 | 0.787 | -11.3 | 8.6 |  |
|  | SHEMI_under vs SHEMI_matched | -16.9 | 0.022* | -31.2 | -2.5 | -0.8 | 0.87 | -9.7 | 8.2 | 7.3 | 0.15 | -2.6 | 17.2 |  |
|  | SHEMI_under vs SHEMI_over | -19.5 | 0.008* | -33.9 | -5.1 | 1.4 | 0.76 | -7.6 | 10.4 | 8.7 | 0.087 | -1.3 | 18.6 |  |
|  | STSA_over vs STSA_matched | -0.8 | 0.91 | -15.2 | 13.5 | -5.3 | 0.244 | -14.3 | 3.6 | -1.1 | 0.823 | -11.1 | 8.8 |  |
|  | STSA_under vs STSA_matched | -2.9 | 0.689 | -17.3 | 11.4 | 1.5 | 0.749 | -7.5 | 10.4 | 0.5 | 0.916 | -9.4 | 10.5 |  |
|  | STSA_under vs STSA_over | -2.1 | 0.775 | -16.5 | 12.3 | 6.8 | 0.137 | -2.2 | 15.8 | 1.7 | 0.742 | -8.3 | 11.6 |  |
|  |  |  |  |  |  |  |  |  |  |  |  |  |  |  |
| **ER** | EHEMI_over vs EHEMI_matched | 7.5 | 0.213 | -4.3 | 19.4 | 1.1 | 0.803 | -7.6 | 9.9 | -7.6 | 0.097 | -16.6 | 1.4 |  |
|  | EHEMI_under vs EHEMI_matched | -1.5 | 0.798 | -13.4 | 10.3 | -0.1 | 0.985 | -8.8 | 8.7 | -4.5 | 0.324 | -13.5 | 4.5 |  |
|  | EHEMI_under vs EHEMI_over | -9.1 | 0.133 | -20.9 | 2.8 | -1.2 | 0.788 | -10.0 | 7.6 | 3.1 | 0.501 | -5.9 | 12.1 |  |
|  | ETSA_over vs ETSA_matched | 7.0 | 0.247 | -4.8 | 18.8 | -4.85 | 0.278 | -13.6 | 3.9 | 0.9 | 0.847 | -8.1 | 9.9 |  |
|  | ETSA_under vs ETSA_matched | 7.3 | 0.227 | -4.5 | 19.1 | 2.2 | 0.623 | -6.6 | 11.0 | 2.1 | 0.654 | -6.9 | 11.0 |  |
|  | ETSA_under vs ETSA_over | 0.3 | 0.96 | -11.5 | 12.1 | 7.05 | 0.115 | -1.7 | 15.8 | 1.2 | 0.799 | -7.8 | 10.1 |  |
|  | SHEMI_over vs SHEMI_matched | 3.8 | 0.528 | -8.0 | 15.7 | -2.5 | 0.579 | -11.2 | 6.3 | -0.3 | 0.948 | -9.3 | 8.7 |  |
|  | SHEMI_under vs SHEMI_matched | -9.2 | 0.126 | -21.1 | 2.6 | -2.4 | 0.589 | -11.2 | 6.3 | 1.7 | 0.719 | -7.3 | 10.6 |  |
|  | SHEMI_under vs SHEMI_over | -13.1 | 0.031* | -24.9 | -1.2 | 0.1 | 0.988 | -8.7 | 8.8 | 2.0 | 0.67 | -7.0 | 10.9 |  |
|  | STSA_over vs STSA_matched | -1.8 | 0.762 | -13.7 | 10.0 | -5.1 | 0.251 | -13.9 | 3.6 | 3.3 | 0.478 | -5.7 | 12.2 |  |
|  | STSA_under vs STSA_matched | -4.0 | 0.505 | -15.9 | 7.8 | -1.4 | 0.76 | -10.1 | 7.4 | 0.8 | 0.858 | -8.2 | 9.8 |  |
|  | STSA_under vs STSA_over | -2.2 | 0.716 | -14.0 | 9.6 | 3.8 | 0.4 | -5.0 | 12.5 | -2.4 | 0.595 | -11.4 | 6.5 |  |
|  |  |  |  |  |  |  |  |  |  |  |  |  |  |  |
| **IR** | EHEMI_over vs EHEMI_matched | 2.6 | 0.578 | -6.7 | 11.9 | -3.2 | 0.409 | -10.9 | 4.4 | -3.3 | 0.404 | -11.1 | 4.5 |  |
|  | EHEMI_under vs EHEMI_matched | -1.6 | 0.738 | -10.9 | 7.7 | -6.2 | 0.11 | -13.9 | 1.4 | -3.7 | 0.354 | -11.4 | 4.1 |  |
|  | EHEMI_under vs EHEMI_over | -4.2 | 0.374 | -13.5 | 5.1 | -3.0 | 0.439 | -10.7 | 4.6 | -0.4 | 0.926 | -8.1 | 7.4 |  |
|  | ETSA_over vs ETSA_matched | 1.8 | 0.712 | -7.5 | 11.0 | 1.4 | 0.716 | -6.2 | 9.1 | -1.1 | 0.781 | -8.9 | 6.7 |  |
|  | ETSA_under vs ETSA_matched | -2.7 | 0.571 | -12.0 | 6.6 | -2.7 | 0.494 | -10.3 | 5.0 | -2.5 | 0.527 | -10.3 | 5.3 |  |
|  | ETSA_under vs ETSA_over | -4.4 | 0.35 | -13.7 | 4.9 | -4.1 | 0.295 | -11.7 | 3.6 | -1.4 | 0.723 | -9.2 | 6.4 |  |
|  | SHEMI_over vs SHEMI_matched | -1.2 | 0.803 | -10.5 | 8.1 | 0.3 | 0.932 | -7.3 | 8.0 | -1.1 | 0.787 | -8.8 | 6.7 |  |
|  | SHEMI_under vs SHEMI_matched | -7.6 | 0.109 | -16.9 | 1.7 | 1.7 | 0.669 | -6.0 | 9.3 | 5.7 | 0.153 | -2.1 | 13.4 |  |
|  | SHEMI_under vs SHEMI_over | -6.4 | 0.176 | -15.7 | 2.9 | 1.3 | 0.732 | -6.3 | 9.0 | 6.7 | 0.089 | -1.0 | 14.5 |  |
|  | STSA_over vs STSA_matched | 1.0 | 0.833 | -8.3 | 10.3 | -0.2 | 0.959 | -7.8 | 7.4 | -4.4 | 0.268 | -12.1 | 3.4 |  |
|  | STSA_under vs STSA_matched | 1.1 | 0.816 | -8.2 | 10.4 | 2.8 | 0.467 | -4.8 | 10.5 | -0.3 | 0.943 | -8.0 | 7.5 |  |
|  | STSA_under vs STSA_over | 0.1 | 0.983 | -9.2 | 9.4 | 3.0 | 0.437 | -4.6 | 10.7 | 4.1 | 0.3 | -3.7 | 11.9 |  |
